# Immunohistochemical and molecular evolutionary features of jejunoileal adenocarcinoma unveiled through comparative analysis with colorectal adenocarcinoma

**DOI:** 10.1016/j.neo.2025.101180

**Published:** 2025-05-21

**Authors:** Rei Ishikawa, Hidetaka Yamada, Hirotomo Saitsu, Ryosuke Miyazaki, Juri Takahashi, Rino Takinami, Satoshi Baba, Mitsuko Nakashima, Moriya Iwaizumi, Satoshi Osawa, Hideya Kawasaki, Yoshifumi Arai, Yoshiro Otsuki, Hiroshi Ogawa, Hiroki Mori, Fumihiko Tanioka, Shioto Suzuki, Kazuyo Yasuda, Makoto Suzuki, Haruhiko Sugimura, Kazuya Shinmura

**Affiliations:** a Department of Tumor Pathology, Hamamatsu University School of Medicine, 1-20-1 Handayama, Chuo-ku, Hamamatsu, Shizuoka 431-3192, Japan; b Department of Biochemistry, Hamamatsu University School of Medicine, 1-20-1 Handayama, Chuo-ku, Hamamatsu, Shizuoka 431-3192, Japan; c Department of Diagnostic Pathology, Hamamatsu University Hospital, 1-20-1 Handayama, Chuo-ku, Hamamatsu, Shizuoka 431-3192, Japan; d Department of Clinical Laboratories, Hamamatsu University School of Medicine, 1-20-1 Handayama, Chuo-ku, Hamamatsu, Shizuoka 431-3192, Japan; e Department of Endoscopic and Photodynamic Medicine, Hamamatsu University School of Medicine, 1-20-1 Handayama, Chuo-ku, Hamamatsu, Shizuoka 431-3192, Japan; f NanoSuit Research Laboratory, Division of Preeminent Bioimaging Research, Institute of Photonics Medicine, Hamamatsu University School of Medicine, 1-20-1 Handayama, Chuo-ku, Hamamatsu, Shizuoka 431-3192, Japan; g Department of Pathology, Toyohashi Municipal Hospital, 50 Hachiken-nishi, Aotake-cho, Toyohashi, Aichi 441-8570, Japan; h Department of Pathology, Seirei Hamamatsu General Hospital, 2-12-12 Sumiyoshi, Chuo-ku, Hamamatsu, Shizuoka 430-8558, Japan; i Department of Pathology, Seirei Mikatahara General Hospital, 3453 Mikatahara-cho, Chuo-ku, Hamamatsu, Shizuoka 433-8558, Japan; j Department of Pathology, Hamamatsu Medical Center, 328 Tomitsuka-cho, Chuo-ku, Hamamatsu, Shizuoka 432-8580, Japan; k Division of Pathology, Iwata City Hospital, 512-3 Okubo, Iwata, Shizuoka 438-8550, Japan; l Department of Pathology, Fujieda Municipal General Hospital, 4-1-11 Surugadai, Fujieda, Shizuoka 426-8677, Japan; m Department of Pathology, Shizuoka General Hospital, 4-27-1 Kita-ando, Aoi-ku, Shizuoka, Shizuoka 420-8527, Japan; n Sasaki Institute, 2-2 Kandasurugadai, Chiyoda-ku, Tokyo 101-0062, Japan

**Keywords:** Small bowel cancer, Immunohistochemical analysis, Whole-exome sequencing, Mismatch repair deficient, Cancer evolutionary history

## Abstract

Jejunoileal adenocarcinoma (JIAC) is a rare type of malignancy, the clinicopathological, genetic, and evolutionary characteristics of which have rarely been reported. In this study, 52 patients with JIAC and 182 patients with colorectal adenocarcinoma (CRAC) were recruited. Immunohistochemical analyses using 34 primary antibodies identified a novel subtype, JIAC with enteroblastic differentiation (JIAED). High MUC1 expression and low Cyclin D1 expression were identified as independent poor prognostic markers. Additionally, compared with mismatch repair deficient (dMMR)-CRAC, MSH2/MSH6 loss was more frequently observed in dMMR-JIAC. These results suggested essential molecular differences between JIAC and CRAC. To better understand these differences, we selected three dMMR-JIACs and eight mismatch repair proficient (pMMR)-JIACs and evaluated molecular evolutionary history by multi-regional whole-exome sequencing. Phylogenetic trees constructed for both pMMR-JIAC and dMMR-JIAC were more consistent with a “long trunk–short branches” structure than were those of CRAC, and the variant allele frequency peaks obtained for JIAC were higher than those of CRAC. Moreover, *TP53* and *ARID2* were identified as common driver gene mutations in pMMR-JIAC, arising during early tumorigenesis. Our evolutionary analysis revealed that pMMR-CRAC follows the principle of shifting from Darwinian to neutral evolution, generating intratumoral heterogeneity (ITH). In contrast, our findings on pMMR-JIAC and dMMR-JIAC demonstrate that both remain under Darwinian evolution, even in advanced stages, resulting in lower ITH. In summary, we identified a distinct pathohistological subtype of JIAC and highlighted the unique molecular evolutionary dynamics presented in JIAC, potentially lead to the better management and treatment strategies for patients with JIAC in the future.

## Highlights

• JIAC with enteroblastic differentiation is identified.
• High MUC1 and low Cyclin D1 protein expressions are associated with poor prognosis.
• MSH2 and MSH6 loss are frequently observed in dMMR-JIAC.
• *TP53* and *ARID2* mutations are identified as early driver gene mutations in JIAC.
• JIAC is affected by Darwinian evolution through tumorigenesis to generate lower ITH.

## Introduction

Small bowel cancer (SBC), which consists of the duodenum and jejunoileum, are rare; they account for <1 % of all cancers. Among the cancers, adenocarcinoma is the most prevalent type in the duodenum, jejunum, and ileum. Given the rarity and anatomical location of SBC, this disease is often considered an extension of colorectal cancer (CRC), and indeed, some comparative studies have revealed the similarities and differences between SBC and CRC based on the findings of immunohistochemical and genomic analyses [1,2]. In addition, recent genomic and transcriptomic analyses have revealed the different molecular characteristics of duodenal adenocarcinoma (DAC) and jejunoileal adenocarcinoma (JIAC) [[3], [4], [5]]. These points highlight the importance of distinguishing JIAC from colorectal adenocarcinoma (CRAC) and DAC, however, previous studies of SBC mixed DAC and JIAC. As a first step to address this, a small bowel malignancy project is currently underway in Japan to develop a Japanese classification of JIAC only [6].

Cancer cells accumulate mutations both under selective pressure and neutrally, with some subclones surviving and generation of intratumoral heterogeneity (ITH). Subclones interact with the tumor microenvironment (TME), influencing tumor progression and therapeutic response. The trajectory of this evolutionary process varies across cancer types and individuals, highlighting the importance of evolutionary analysis for optimization of therapeutic strategies and the discovery of new therapeutic targets [7]. Multi-regional genome sequencing has made it possible to infer genetic ITH, which is visualized by phylogenetic trees constructed by cancer cell fractions (CCFs), and distributions of variant allele frequencies (VAFs). Sottoriva *et al*. [8] presented a "Big Bang model" of tumor progression: the tumor grew predominantly as a single expansion and produced numerous intermixed subclones without selection by tumor evolutionary analysis, including phylogenetic tree construction. Saito *et al*. [9] presented a revised model called the evolutionary principle, which included a shift from Darwinian evolution: driver mutations played an important role in the selective sweep of subclones to neutral evolution with passenger mutations accumulated without selection. The clonal structure of the cancer is encoded by the shape of the VAF distribution: high VAF with sharp peaks indicates subclones arising at early stages and expanding rapidly under strong selective pressure, while low VAF with broad peaks suggests subclones arising later and evolving neutrally [10]. Driver gene mutations influence the evolutionary history of cancer: driver gene mutations in early stages boost selective pressure, whereas those in advanced stages contribute to drug resistance and metastasis [11]. Phylogenetic trees, VAF distributions, and driver gene mutations interact closely throughout tumorigenesis, providing critical insights into understanding cancer evolution and ITH. Such evolutionary analysis has been performed for several common cancers, including CRAC [12], however, JIAC has not undergone evolutionary analysis and driver gene detection.

In this study, we aimed to identify the unique clinicopathological, genetic, and evolutionary characteristics of JIAC via its comparative analysis with CRAC using comprehensive immunohistochemical and multi-regional whole-exome sequencing (WES) analysis.

## Material and methods

### Patient cohort and ethics

Fifty-two patients with JIAC were recruited from various hospitals in Japan, including Hamamatsu University Hospital (HUH), Hamamatsu Medical Center, Seirei Hamamatsu General Hospital, Seirei Mikatahara General Hospital, Iwata City Hospital, Fujieda Municipal General Hospital, Shizuoka General Hospital, and Toyohashi Municipal Hospital. Additionally, 182 patients with CRAC were recruited from HUH. All patients underwent surgery to resect primary JIAC or CRAC between 2011 and 2022 and did not receive adjuvant therapies. The Institutional Review Board of Hamamatsu University School of Medicine (HUSM) approved our study (No. 20-011 and 23-348), which was conducted in accordance with the Declaration of Helsinki. The requirement for patient consent was waived due to the retrospective nature of the study, use of anonymized samples from pathologic archives, and implementation of the opt-out procedure through a public information notice.

### Tissue microarray construction and immunohistochemical analysis

A total of 55 JIACs and 192 CRACs were used to construct tissue microarray (TMA) blocks, as previously described [13,14]. They included 3 patients with JIAC and 10 patients with CRAC affected by double adenocarcinoma. TMA blocks were also constructed using paired non-cancerous epithelial tissue of the jejunoileum or colorectum derived from 52 patients with JIAC or 182 patients with CRAC, respectively. Immunohistochemical analysis was performed using a Dako autostainer or VENTANA BenchMark ULTRA platform (VENTANA, RRID:SCR_025506). The primary antibodies and immunohistochemical conditions of the experiment are listed in Supplementary Table S1A. Two pathologists (RI and KS) independently evaluated the protein expressions using the cut-off values also listed in Supplementary Table S1B.

### Field emission-scanning electron microscopy analysis using the NanoSuit-correlative light and electron microscopy

Sections used for villin-1 immunohistochemistry were also used for field emission-scanning electron microscopy (FE-SEM) using the NanoSuit-correlative light and electron microscopy (CLEM) [14]. After 3,3′-diaminobenzidine tetrahydrochloride (DAB) enhancement with 1 % osmium solution, the section was rehydrated by coating with the surface shield enhancer solution. The section was examined with a Hitachi S-4800 FE-SEM (Hitachi, Tokyo, Japan, RRID:SCR_020019).

### DNA isolation and WES analysis

For the WES analysis, 3 mismatch repair deficient (dMMR)-JIACs and 8 mismatch repair proficient (pMMR)-JIACs were selected from 52 JIAC cases. DNA isolation was performed for both cancerous and non-cancerous samples for each case. For pMMR-JIACs, DNA was isolated from multiple regions (i.e., 3-7 locations per adenocarcinoma) for evolutionary analysis. The QIAamp DNA FFPE Advanced UNG Kit (QIAGEN, Limberg, Netherlands) was utilized for DNA isolation. The quantity and quality of double-stranded DNA (dsDNA) were measured using Qubit 2.0 Fluorometer (Thermo Fisher Scientific, RRID:SCR_020553), NanoDrop 1000 (Thermo Fisher Scientific, RRID:SCR_016517), 4150 TapeStation (Agilent Technologies, Santa Clara, CA, USA, RRID:SCR_019393). All DNA samples met the following conditions: (a) dsDNA concentration of ≥ 50 ng/㎕; (b) Optical Density (OD) 260/280 ≥ 1.8 and OD 260/230 ≥ 1.6; and (c) DNA integrity number (DIN) of ≥ 2.5.

The DNA libraries were prepared using Twist Library Preparation Enzymatic Fragmentation Kit 2.0 and captured with Exome 2.0 Panel (TwistBioscience, South San Francisco, CA, USA). The captured libraries were sequenced with 100 bp pair-end reads, ≥ 200x depth for tumor samples, and ≥ 100x depth for normal samples on DNBSEQ-G400 (MGI, Shenzhen, China, RRID:SCR_017980). Low quality bases and adaptors were removed from the raw reads using Fastp (v0.21.0, RRID:SCR_016962) (https://github.com/OpenGene/fastp) [15]. Cleaned reads were mapped to the human reference genome (GRCh38decoyJRGv2) (https://jmorp.megabank.tohoku.ac.jp) [16] using BWA-MEM (v0.7.17, RRID:SCR_010910) (https://github.com/lh3/bwa) [17], followed by compression using SAMtools (v1.10, RRID:SCR_002105) (https://github.com/samtools) [18]. PCR duplicates were removed and base quality scores were recalibrated using GATK4 (v4.1.9.0, RRID:SCR_001876) (https://github.com/broadinstitute/gatk) [19].

### Chromosomal copy number variant analysis and calculation of tumor purity and ploidy

Chromosomal copy number variant (CNV) analysis and calculation of tumor purity and ploidy were performed using FACETS (v.0.5.14) (https://github.com/dariober/cnv_facets) [20]. Loss of heterozygosities (LOHs) were identified using Varscan2 (v.2.4.6) (https://github.com/dkoboldt/varscan) [21].

### Evaluation of microsatellite instability status and calculation of tumor mutation burden

Microsatellite instability (MSI) scores were calculated for tumor samples with WES using MSIsensor-pro (v.1.2.0, RRID:SCR_024765) (https://github.com/xjtu-omics/msisensor-pro) [22] to determine if they were MSI/dMMR tumors. The tumor mutation burden (TMB) was also calculated using Maftools (v.2.14.0, RRID:SCR_024519) (https://github.com/PoisonAlien/maftools) [23]. MSI/dMMR was defined as an MSI score of > 3.5, and high TMB was defined as ≥ 10 mutations/megabase (Mb).

### SNV analysis and driver gene identification

Somatic SNVs were identified using Mutect2 in GATK4 [19], Strelka2 (v.2.9.10) (https://github.com/Illumina/strelka) [24], and Varscan2 [21] with a minimum VAF of 0.10. An SNV was considered true only if it was identified by all three callers. ANNOVAR (v.20200608, RRID:SCR_012821) (https://github.com/WGLab/doc-ANNOVAR) [25] was used to annotate the SNVs. Maftools were used to construct oncoplots and identify the top 20 frequently mutated genes [23]. Driver genes were identified using SNV data, OncodriveFML (v.2.3.0) (https://bitbucket.org/bbglab/oncodrivefml.git/src) [26], and dNdScv (v.0.0.1.0, RRID:SCR_017093) (https://github.com/im3sanger/dndscv) [27]. Genes obtained from both methods (*P* < 0.05) were selected as candidates for driver genes. For the germline SNVs, they were identified using HaplotypeCaller and GenotypeGVCFs in GATK4 [19]. ANNOVAR [25] was also used to annotate the SNVs.

### Phylogenetic tree construction

Phylogenetic trees were constructed using our WES analysis data for JIAC and Meskit dataset for CRAC [28] realigned to GRCh38 using CrossMap (v.0.6.5, RRID:SCR_001173) (https://github.com/liguowang/CrossMap) [29]. Public raw data (ID: SRP252694) for CRAC from the Sequence Read Archive (SRA) database (RRID:SCR_004891) (https://www.ncbi.nlm.nih.gov/sra) [30] were used for phylogenetic tree construction. The CCFs of the JIACs and CRACs were estimated using PyClone-vi (v.0.1.2) (https://github.com/Roth-Lab/pyclone-vi) [31] under the following conditions: a maximum of 40 clusters, 100 random restarts, and binomial probability density. The phylogenetic trees were constructed using the neighbor-joining method, CCF data, and MesKit (v.1.8.0, RRID:SCR_020959) (https://github.com/Niinleslie/MesKit) [32]. The driver gene information for JIAC mentioned in the previous paragraph and that of CRAC collected from the IntOGen database (https://www.intogen.org) [33] were used to annotate the tree.

### VAF distribution analysis

The distribution of VAFs was analyzed using Maftools [23]. The VAF distribution has several peaks (density > 2.0), including a clonal peak (theoretical VAF = 0.5), a subclonal peak (VAF < 0.5), and a neutral tail (VAF close to 0) associated with the accumulation of passenger mutations as the tumor expands [10]. Since sequencing methods, tumor purity, and tumor ploidy can affect these peaks, this study used the following thresholds: clonal peak, VAF = 0.40–0.50; subclonal peak, VAF = 0.15–0.40; and neutral tail, VAF < 0.15. The study also calculated the mutant-allele tumor heterogeneity (MATH) score, which is the ratio of the width to the center of the distribution of VAFs among tumor-specific mutated loci [34] to quantify the distributions.

### Statistical analysis

Pearson's chi-squared test, Student's *t*-test, Fisher's exact test, Mann-Whitney *U*-test, log-rank test, tetrachoric correlation analysis, Cox's univariate and multivariate analyses, and plot constructions were conducted using IBM SPSS Statistics (v.27, RRID:SCR_016479) and R packages. Statistical significance was denoted by *P*-values of < 0.05 or *Q*-values of < 0.05.

## Results

### Clinicopathologic features detected in patients with JIAC

We collected data from 52 Japanese patients with JIAC and 182 Japanese patients with CRAC who underwent surgery for carcinoma in Japan. We also examined their clinicopathologic features. Among the 13 clinicopathological parameters analyzed, age, sex assigned at birth, abdominal symptoms, tumor size, gross tumor type, and histopathological type showed significant differences for the two groups. JIAC had a lower age of onset; was more prevalent in males; and presented with more frequent abdominal symptoms, a larger tumor size, and a higher frequency of por/muc histopathology than CRAC (Supplementary Table S2A). These results suggest that patients with JIAC have clinicopathological differences from those of patients with CRAC.

### Characteristics of immunohistochemical protein expression in JIAC

**Fig. 1:**
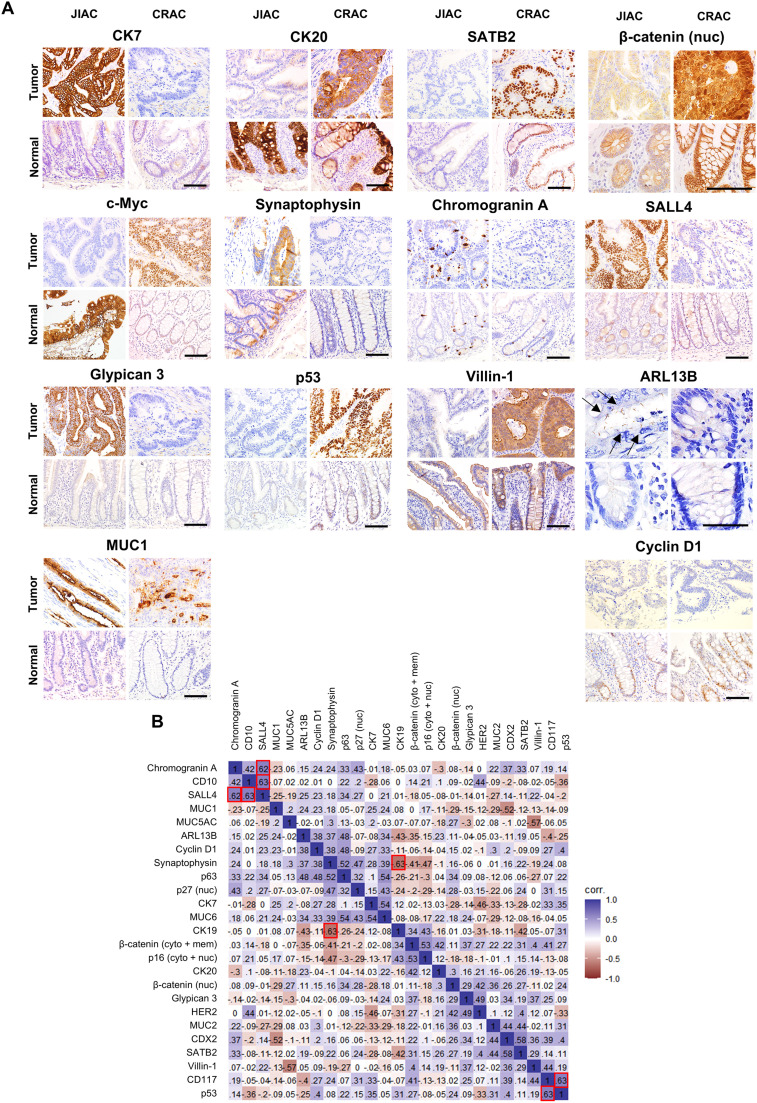
**Protein expressions of JIAC and CRAC by comprehensive immunohistochemical analysis. (A)** Representative immunohistochemical images of JIAC (top left) and CRAC (top right). The corresponding non-cancerous areas of the jejunoileum and colorectum are also illustrated (bottom left and bottom right, respectively). From the 32 proteins analyzed in this study, those with statistically significant different expressions in JIAC and CRAC (Student's *t*-test or Fisher's exact test; *P* < 0.05) or different prognoses associated with high and low expressions in JIAC (log-rank test; *P* < 0.05) were selected in these panels. Please refer to **Fig. 3B** for representative images of mismatch repair-related proteins. Arrows indicate primary cilia. Scale bar, 50 μm for ARL13B; 100 µm for the others. **(B)** Tetrachoric correlation model of protein expression in JIAC. All JIACs were scored for high or low expression for each protein, and all results except CK17, BRAF V600E, AFP, Oct3/4, and SOX2, which were excluded because their expressions were high or low only during their immunohistochemical analysis, were subjected to tetrachoric correlation analysis. The tetrachoric correlation coefficient values above 0.60 or below −0.60 were represented by a red square. When the same items were compared in this tetrachoric correlation analysis, the value was shown as 1.0.

Using FFPE blocks derived from the patients with JIAC and CRAC, TMA blocks were prepared for 55 tumors of JIACs (from 52 patients) and 192 tumors of CRACs (from 182 patients). Immunohistochemical analysis was performed using 32 primary antibodies commonly used in diagnostic pathology. The following statistically differential expressions were observed for JIAC and CRAC. JIAC was associated with higher expression of CK7, synaptophysin, chromogranin A, SALL4, glypican 3, and ARL13B than CRAC. However, the expressions of CK20, SATB2, β-catenin (nuc), c-Myc, p53, and villin-1 were lower for JIAC than for CRAC (Table 1 and Fig. 1A). The subsequent section will present the findings for the MMR-related protein. Of the aforementioned 12 markers, the expression levels of 9 in JIAC and non-cancerous epithelial tissues differed (Supplementary Table S3A), suggesting that most of the JIAC-specific immunohistochemical features were tumor-specific.

**Table 1:** Comparison of immunohistochemical expressions of JIAC and CRAC.

Immunohistochemical marker	Expression status	Number of cases (%)		*P*-value a
		JIAC	CRAC	
Cytokeratin				
CK7	high	13 (25.0)	11 (5.8)	**< 0.001**
	low	39 (75.0)	179 (94.2)	
CK17	high	0 (0.0)	0 (0.0)	NULL
	low	52 (100.0)	190 (100.0)	
CK19	high	46 (88.5)	149 (78.4)	0.105
	low	6 (11.5)	41 (21.6)	
CK20	high	30 (56.6)	149 (78.4)	**0.001**
	low	23 (43.4)	41 (21.6)	
Mucin and intestinal marker				
MUC1 (cyto + mem)	high	14 (26.9)	54 (28.6)	0.815
	low	38 (73.1)	135 (71.4)	
MUC2	high	26 (50.0)	82 (43.2)	0.379
	low	26 (50.0)	108 (56.8)	
MUC5AC	high	11 (21.2)	25 (13.2)	0.151
	low	41 (78.8)	165 (86.8)	
MUC6	high	2 (3.8)	3 (1.6)	0.295
	low	50 (96.2)	186 (98.4)	
CD10	high	9 (17.3)	52 (27.4)	0.139
	low	43 (82.7)	138 (72.6)	
CDX2	high	33 (61.1)	124 (64.9)	0.785
	low	21 (38.9)	67 (35.1)	
SATB2	high	32 (61.5)	155 (82.0)	**0.002**
	low	20 (38.5)	34 (18.0)	
MMR-related protein				
MLH1	absence	2 (3.8)	18 (9.6)	0.260
	presence	50 (96.2)	169 (90.4)	
PMS2	absence	3 (4.9)	17 (10.2)	0.421
	presence	48 (94.1)	150 (89.8)	
MSH2	absence	7 (13.5)	1 (0.6)	**< 0.001**
	presence	45 (86.5)	175 (99.4)	
MSH6	absence	7 (13.5)	1 (0.5)	**< 0.001**
	presence	45 (86.5)	183 (99.5)	
Any of the four MMR proteins above	absence	10 (19.2)	19 (10.7)	0.102
	presence	42 (80.8)	159 (89.3)	
BRAF V600E	presence	0 (0.0)	5 (2.6)	0.588
	absence	52 (100.0)	185 (97.4)	
Wnt signaling pathway				
β-catenin (cyto + mem)	high	44 (84.6)	148 (78.3)	0.317
	low	8 (15.4)	41 (21.7)	
β-catenin (nuc)	high	5 (9.6)	80 (42.3)	**< 0.001**
	low	47 (90.4)	109 (57.7)	
CD117	high	31 (57.4)	99 (52.4)	0.514
	low	23 (42.6)	90 (47.6)	
c-Myc	high	2 (3.8)	77 (40.5)	**< 0.001**
	low	50 (96.2)	113 (59.5)	
Cyclin D1	high	46 (88.5)	149 (78.4)	0.105
	low	6 (11.5)	41 (21.6)	
Neuroendocrine marker				
Synaptophysin	high	2 (3.8)	0 (0.0)	**0.046**
	low	50 (96.2)	189 (100.0)	
Chromogranin A	high	4 (7.7)	0 (0.0)	**0.002**
	low	48 (92.3)	189 (100.0)	
Enteroblastic marker				
SALL4	high	4 (7.7)	1 (0.5)	**0.008**
	low	48 (92.3)	188 (99.5)	
Glypican 3	high	11 (21.2)	2 (1.1)	**< 0.001**
	low	41 (78.8)	184 (98.9)	
AFP	high	0 (0.0)	0 (0.0)	NULL
	low	52 (100.0)	190 (100.0)	
Transcriptional factor and others				
p16 (cyto + nuc)	high	16 (30.8)	40 (21.1)	0.141
	low	36 (69.2)	150 (78.9)	
p27 (nuc)	high	22 (40.7)	67 (35.3)	0.461
	low	32 (59.3)	123 (64.7)	
p53	high	13 (25.0)	76 (40.0)	**0.047**
	low	39 (75.0)	114 (60.0)	
p63	high	1 (1.9)	1 (0.5)	0.397
	low	53 (98.1)	187 (99.5)	
HER2	high	1 (1.9)	3 (1.6)	1.000
	low	51 (98.1)	187 (98.4)	
Villin-1	high	36 (69.2)	156 (83.4)	**0.023**
	low	16 (30.8)	31 (16.6)	
ARL13B	high	2 (3.8)	0 (0.0)	**0.046**
	low	50 (96.2)	189 (100.0)	

cyto, cytoplasm; mem, membrane; nuc, nucleus; MMR, mismatch repair.

a *P* values calculated by using Pearson's chi-square test or Fisher's exact test. Significant values (i.e. *P* < 0.05) are shown in bold.

Of the above immunohistochemical findings, we considered the following to be of particular importance. First, 15 tumors of JIAC were identified with enteroblastic differentiation, as demonstrated by the enteroblastic lineage markers SALL4 and glypican 3 (Table 1 and Fig. 1A). The disease was designated as JIAC with enteroblastic differentiation (JIAED), which serves as a counterpart to CRAC with enteroblastic differentiation (CRAED) [35]. When comparing JIAED to non-JIAED, no significant clinical or histological differences were observed, except for location: JIAED was preferentially located in jejunum (Supplementary Table S2B). Interestingly, two (50 %) of four patients with JIAED were also found to be positive for chromogranin A, and a strong correlation between SALL4 expression and chromogranin A expression in JIAC was identified by tetrachoric correlation analysis (Fig. 1B). Second, SATB2 and villin-1 are immunohistochemical markers for CRAC [36]; however, they were significantly less positive for JIAC (SATB2, 61.5 %; villin-1, 69.2 %) than for CRAC (SATB2, 82.0 %; villin-1, 83.4 %) (Table 1). Villin-1 is a component of microvilli, and they were observed with FE-SEM analysis using the NanoSuit-CLEM method. The microvilli in JIAC were fewer than those in CRAC (Supplementary Fig. S1). This represents one of the morphological distinctions between JIAC and CRAC. Third, two JIACs with primary cilia were identified, as shown by ARL13B immunostaining (Table 1 and Fig. 1A). Fourth, the distribution of the CK7/CK20 immunophenotype was significantly different (*P* < 0.001), and the CK7(-)/CK20(+) type, which was the most frequent (74.2 %) in CRAC, was less frequent (42.3 %) in JIAC (Supplementary Table S3B).

**Fig. 2:**
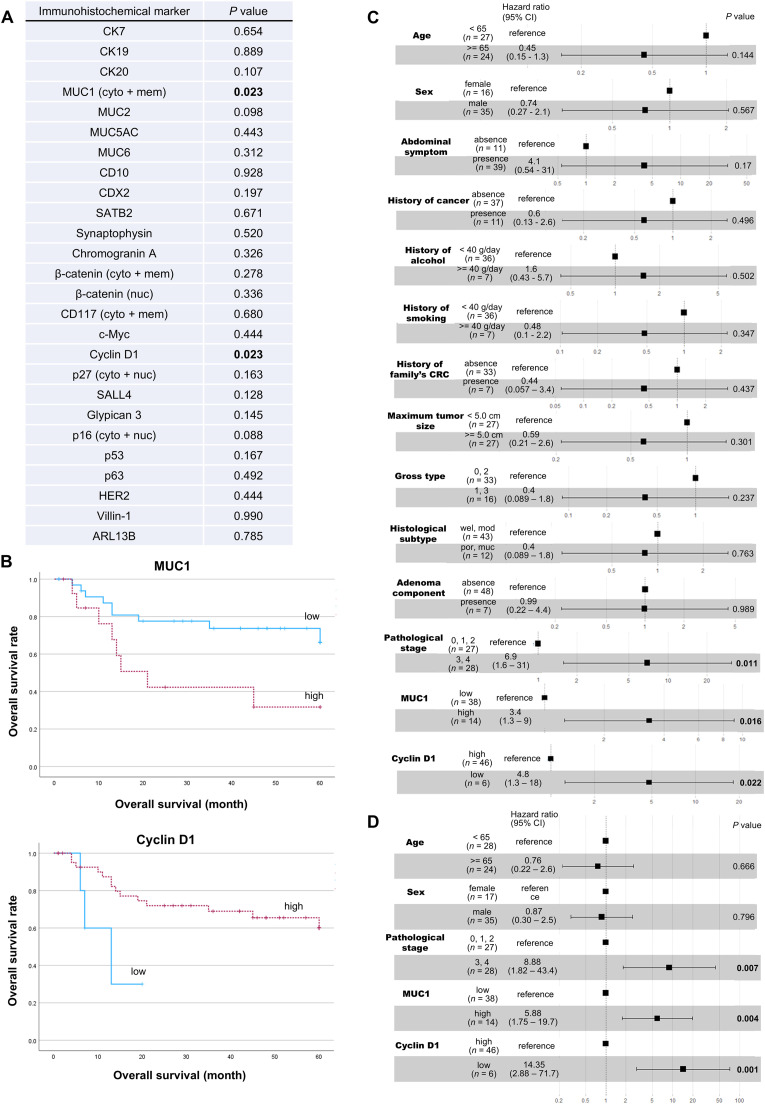
**Impact of protein expression on overall survival of patients with JIAC. (A)** List of *P*-values obtained by the log-rank test for the survival curve constructed by the Kaplan-Meier method. For each protein, patients with JIAC were divided into the higher and lower expression groups, and Kaplan–Meier analysis was performed. *P*-values are shown for all proteins except CK17, BRAF V600E, AFP, Oct3/4, and SOX2, which were excluded because their expressions were high or low only during their immunohistochemical analysis. Bold indicates statistical significance (*P* < 0.05). Cyto: cytoplasmic staining; mem: membranous staining; nuc: nuclear staining. **(B)** Effect of MUC1 and cyclin D1 expressions on overall survival of patients with JIAC. The Kaplan-Meier survival curve was constructed, and the calculated *P*-values are shown in **A. (C)** Cox's univariate analysis of potential predictors including MUC1 and cyclin D1 expressions associated with poor prognosis of JIAC. **(D)** Cox's multivariate analysis of potential predictors including MUC1 and cyclin D1 expressions in JIAC with poor prognosis.

Twenty-six immunohistochemical markers were analyzed for their predictive values for the prognosis of JIAC. Kaplan–Meier analysis showed that the overall survival of patients with JIAC with high MUC1 protein expression was significantly shorter than that of patients with JIAC with low MUC1 expression. Additionally, that of the patients with JIAC with low cyclin D1 expression was significantly worse than that of the patients with JIAC with high cyclin D1 expression (Figs. 2A and B). Cox's univariate (Fig. 2C) and multivariate (Fig. 2D) analyses for overall survival showed that the expressions of MUC1 and cyclin D1 were associated with significantly increased risk of poor overall survival, hazard ratio (HR) of univariate analysis, 3.4 [95 % confidence interval (CI), 1.3–9; *P* = 0.016] for MUC1 and 4.8 (95 % CI, 1.3–18; *P* = 0.022) for cyclin D1; HR of multivariate analysis, 5.88 (95 % CI, 1.75–19.7; *P* = 0.004) for MUC1 and 14.35 (95 % CI, 2.88–71.7; *P* = 0.001) for cyclin D1. These results suggest that high MUC1 and low cyclin D1 expressions are independent predictors of poor survival in patients with JIAC.

### Characterization of dMMR-JIAC

**Fig. 3:**
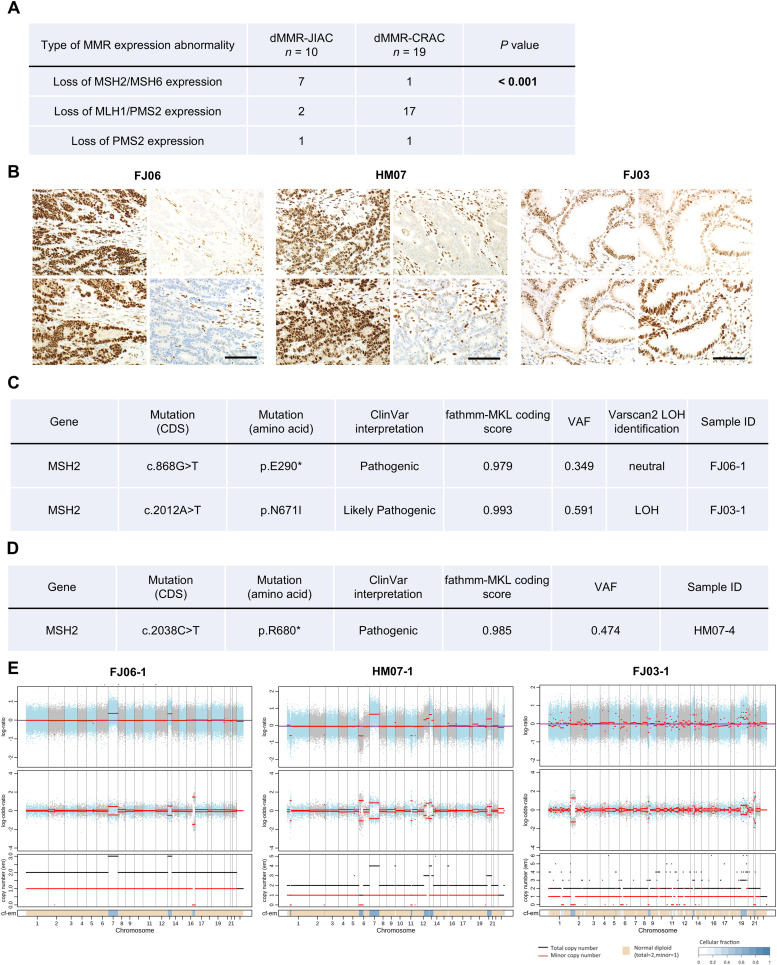
**Immunohistochemical and genetic characteristics of dMMR-JIAC. (A)** Comparison of immunohistochemical expression profiles of four MMR proteins in dMMR-JIAC (*n* = 10) and dMMR-colorectal adenocarcinoma (*n* = 19). Pearson's chi-squared test was performed. (**B)** Representative immunohistochemical images of four MMR proteins in two patients with dMMR-JIAC (FJ06 and HM07) are shown: MLH1 (top left), PMS2 (bottom left), MSH2 (top right), and MSH6 (bottom right). In these patients, the expressions of both MSH2 and MSH6 were lost, while those of both MLH1 and PMS2 were retained. The losses of MSH2 and MSH6 expression in both patients were cancer-cell specific. Patient FJ03 is also shown as an irregular case. This case demonstrated the expression of all four MMR proteins. However, WES analysis revealed high MSI score and TMB, which are consistent with the dMMR phenotype (Supplementary Table S3A). Scale bar, 100 µm. **(C)** Detection of somatic point mutations in *MSH2*, one of the MMR genes, and the corresponding chromosomal copy number status for two dMMR-JIACs (FJ06 and FJ03). The mutations were annotated using ANNOVAR, including ClinVar interpretation and fathmm-MKL coding score of the mutations were used to ascertain their pathogenicity. The LOHs were detected using Varscan2. **(D)** Detection of the germline *MSH2* mutation in a dMMR-JIAC case (HM07). It was identified using HaplotypeCaller and GenotypeGVCFs in GATK4. It was also annotated using ANNOVAR. **(E)** Chromosomally stable phenotype of three patients with dMMR-JIAC (FJ06, HM07, and FJ03) demonstrated by integrated visualization of FACETS analysis of WES data. The top panel shows the log ratio of the total chromosome copy number. The green line indicates the median log ratio for the sample. The purple line indicates the log ratio of the diploid state. The second panel shows the allele-specific log-odds ratio. The segment means are plotted as red lines. The third panel shows the total (black) and minor (red) copy number (em) for each segment. The lower bar shows the corresponding cellular fraction (cf).

dMMR is an important phenotype of a subset of CRAC [37], dMMR-JIACs were searched for and characterized by immunohistochemical and WES analysis. Of the 52 JIACs, 10 dMMR-JIACs (19.2 %) were identified by immunohistochemical analysis using primary antibodies against MMR proteins (i.e., MLH1, MSH2, PMS2, and MSH6) with an almost similar frequency in CRAC (19/178, 10.7 %) (Table 1). However, the loss of expressions of both MSH2 and MSH6 were observed significantly more often in dMMR-JIAC (7/10, 70.0 %) than in dMMR-CRAC (1/19, 5.3 %) (*P* < 0.001) (Figs. 3A and B), suggesting that the loss of MSH2/MSH6 may be a feature of dMMR-JIAC. In addition, most of them lacked tumor-infiltrating lymphocytes and maintained tubule forming, well-known features of dMMR-CRAC (Fig. 3B). Therefore, WES analysis was performed for the two dMMR-JIACs with MSH2/MSH6 loss. One patient (FJ06) had a somatic *MSH2* mutation (p.E290*), and the other patient (HM07) had a germline *MSH2* mutation (p.R680*) (Figs. 3C and D). The family of the latter was fulfilled the new Amsterdam Criteria and was diagnosed with Lynch syndrome (Supplementary Table S3C) [38]. WES analysis of a JIAC patient (FJ03) that retained 4 MMR expressions but was determined to be dMMR based on the MSI score and TMB (Fig. 3A and Supplementary Table S4A) revealed a somatic pathogenic *MSH2* missense mutation (p.N671I) and LOH at the *MSH2* locus (Fig. 3C). This suggested that the *MSH2* abnormality caused the dMMR phenotype in this case. In addition, the chromosomally stable phenotype detected in these three dMMR-JIACs (Fig. 3E) is consistent with the chromosomally stable phenotype commonly observed in dMMR-CRAC [39]. Based on the above results, we concluded that the majority of dMMR-JIACs are due to the loss of MSH2/MSH6.

### Molecular evolutionary history of JIAC

**Fig. 4:**
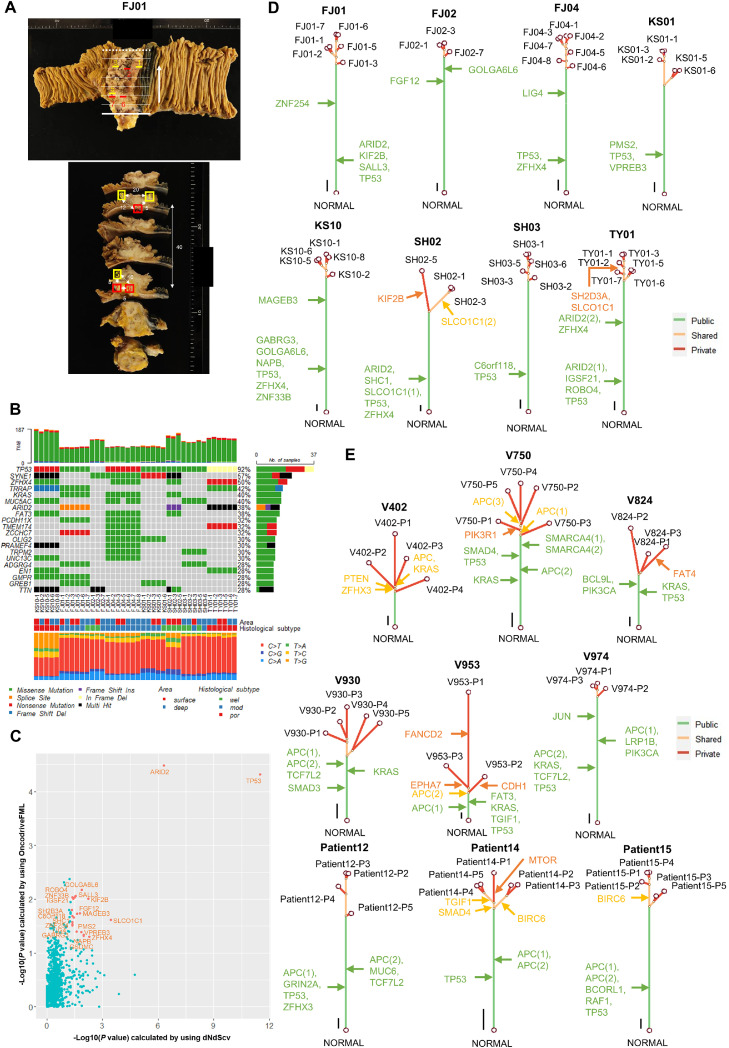
**Phylogenetic trees of pMMR-JIACs and pMMR-CRACs. (A)** A representative map of sampling locations for pMMR-JIAC (patient ID: FJ01). The upper panel shows the section lines. Each line is approximately 10 mm apart. After sectioning, each sample was tilted at right angles in the direction of the arrow. The sampling location is indicated by yellow or red lines and a serial number is added to each location. Yellow indicates that the sample was taken from the mucosal to the submucosal layer, whereas red indicates that the sample was taken from a layer deeper than the muscularis propria. The lower panel also shows the sampling location on the section surface. Yellow and red mean the same in the upper panel. Two close sampling locations are connected by a double-headed arrow, and the distance (mm) is shown on the cut surface. The other maps showing the sampling locations of pMMR-JIAC are shown in Supplementary Fig. S2. **(B)** Somatic mutation landscape of 40 tumor samples derived from 8 pMMR-JIACs. This oncoplot panel was generated by Maftools. The top 20 most frequently mutated genes, ranked by their frequency, are included in this oncoplot. The TMB (total number of somatic mutations), sampling area (surface and deep mean mucosal to submucosal layer and layer deeper than muscularis propria, respectively), histological subtype (well to poorly differentiated adenocarcinoma), and six classes of base substitution (%) are also shown. **(C)** Identification of driver genes of pMMR-JIAC using dNdScv and OncodriveFML. Total mutations of 40 tumor samples derived from 8 pMMR-JIACs were subjected to dNdScv or OncodriveFML to search for candidate genes with cancer driver mutations. Significance threshold (*P* = 0.05) corresponds to -log_10_(*P*-value) = 1.301. Genes with significant detection in both dNdScv and OncodriveFML were selected as candidate driver genes. **(D)** Phylogenetic trees of 8 pMMR-JIACs. Mutation data were detected by multi-regional WES of pMMR-JIACs. All somatic point mutation data (consisting of data for base substitutions and small insertions/deletions) were subjected to PyClone-vi, and CCFs per tumor were estimated. Phylogenetic trees were constructed by neighbor-joining using the CCF data. Mutations are classified into three groups: public (observed in all multi-regional samples; green), private (observed in only one sample; red), and shared (neither public nor private; yellow). Driver gene mutations were identified from all SNVs in pMMR-JIAC and the occurrence of them were annotated with an arrow. Detailed data are shown in Supplementary Tables S5C. Scale bar, 10 mutations. **(E)** Phylogenetic trees of 9 pMMR-CRACs. Mutation data were obtained from Meskit dataset and detected by multi-regional WES of public WES data (ID: SRP252694) from the SRA database. Detailed data are shown in Supplementary Tables S5D. Scale bar, 10 mutations.

The differences in immunohistochemical expression between JIAC and CRAC suggest evolutionary genetic distinctions between these two entities, prompting further investigation. First, we selected eight cases classified as pMMR-JIAC based on immunohistochemical analysis (Table 1), for which DNA quality was sufficient to perform WES. A total of 40 tumor and 8 normal DNA samples (3–7 tumor samples and one normal sample per case) were selected for the analysis (Fig. 4A and Supplementary Fig. S2). All samples were confirmed as pMMR based on the MSI score and TMB, and high tumor purity (≥ 0.40) (Supplementary Table S4B).

Before construction of phylogenetic trees, we performed SNV identification, annotation, and driver gene identification. The top 8 frequent SNVs were as follows: *TP53, SYNE1, ZFHX4, TRRAP, KRAS, MUC5AC, ARID2,* and *FAT3*. Most of them were common in each patient regardless of the sampling area and histopathologic subtypes (Fig. 4B). We used dNdScv and OncodriveFML to identify 22 candidate driver genes of JIAC, including *TP53* and *ARID2* but excluding *APC* and *KRAS*; these are common driver genes in CRAC [40] (Fig. 4C). These results suggested that the carcinogenesis of JIAC was different from that of CRAC in terms of driver gene mutations. In addition, *ARID2* was identified as the most significant driver gene in JIAED, not identified in non-JIAED (Supplementary Figs. S3A and B).

**Figure 5:**
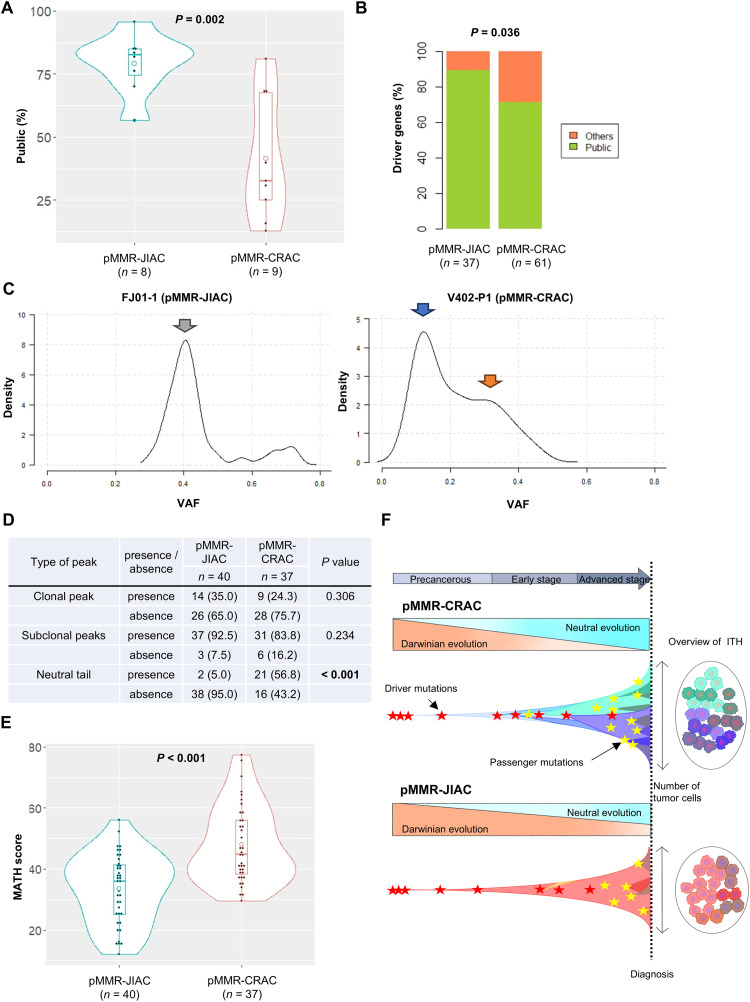
**Comparison of the characteristics of the molecular evolutionary histories of pMMR-JIAC and pMMR-CRAC.** The mutation data of pMMR-JIAC were detected via our multi-regional WES analysis, while the mutation data of pMMR-CRAC were obtained from Meskit dataset and detected by public WES data (ID: SRP252694) from the SRA database. **(A)** Comparison of the proportions of public mutations observed in phylogenetic trees of pMMR-JIAC and pMMR-CRAC. The proportions of 8 pMMR-JIACs and 9 pMMR-CRACs were analyzed using violin boxplots (Mann-Whitney *U*-test). The violin plot shows the relative frequency of each value represented by dots, while the boxplot shows the median and interquartile range. The white circle indicates the mean. **(B)** Comparison of the frequencies of public driver gene mutations observed in phylogenetic trees of pMMR-JIAC and pMMR-CRAC. The frequency was significantly different for pMMR-JIACs (number of driver gene mutations is 37) and pMMR-CRACs (number of driver gene mutations is 61) (89.5 % vs. 71.7 %, Pearson's chi-squared test). **(C)** Representative data of the VAF distribution of pMMR-JIAC and pMMR-CRAC plotted using the Mclust method. The left panel is derived from a region of pMMR-JIAC (sample ID: FJ01-1), while the right panel is derived from a region of pMMR-CRAC (sample ID: V402-P1). Each peak (density > 2.0) in the VAF distribution was classified as a clonal peak (VAF = 0.40-0.50; grey arrow), subclonal peak (VAF = 0.15-0.40; orange arrow), or neutral tail (VAF = 0-0.15; blue arrow). **(D)** Comparison of the proportions of peaks in the VAF distributions of pMMR-JIAC and pMMR-CRAC. Peak data were analyzed by Pearson's chi-squared test between 40 tumor samples derived from 8 pMMR-JIACs and 37 tumor samples derived from 9 pMMR-CRACs. Bold font, *P* < 0.05. **(E)** Comparison of the distributions of MATH scores based on VAF data for pMMR-JIAC and pMMR-CRAC. The distribution of MATH scores in 40 tumor samples derived from 8 pMMR-JIACs and 37 tumor samples derived from 9 pMMR-CRACs were analyzed by violin boxplots (Mann-Whitney *U*-test). **(F)** Schematic representation of the proposed evolutionary model of the pMMR-JIAC in comparison with pMMR-CRAC. In pMMR-CRAC, driver gene mutations generate multiple subclones at an early stage, but only a few subclones survive through Darwinian evolution. During tumor progression, passenger mutations accumulate neutrally in the tumor (neutral evolution). In other words, pMMR-CRAC maintains ITH by shifting evolutionary principle. On the other hand, in pMMR-JIAC, Darwinian evolution continues to affect the tumor, even at advanced stages, although neutral evolution is present. This finding suggests that pMMR-JIAC undergoes stronger and more continuous selective pressure, leading to lower ITH compared to pMMR-CRAC.

Next, we constructed phylogenetic trees for our 8 advanced pMMR-JIACs using multi-regional WES data, as well as for 9 advanced pMMR-CRACs, utilizing data from the Meskit dataset and the SRA database (Figs. 4D and E; Supplementary Tables S5A and B; the pT stage did not show a significant difference between the two groups). Mutations utilized for the construction of phylogenetic trees were categorized as public (observed in all samples of a tumor), shared (observed in a few samples of a tumor), or private (observed in only a single sample of a tumor). In addition, driver gene mutations were mapped along the phylogenetic trees (Supplementary Tables S5C and D). When classifying phylogenetic tree shapes as "long trunk-short branches" (i.e., predominance of public mutations) and "short trunk-long branches" (i.e., predominance of shared and/or private mutations) [9], all pMMR-JIACs exhibited a "long trunk-short branches" pattern. Similarly, all nine pMMR-CRACs were categorized as "long trunk-short branches"; however, two cases displayed an atypical pattern, characterized by a "relatively long trunk rather than a short trunk and relatively short branches rather than long branches." This distinction suggests subtle differences in the evolutionary dynamics between pMMR-JIACs and pMMR-CRACs. Upon further analysis of the mutations constituting the phylogenetic trees, public mutations were more common in the 8 pMMR-JIACs than in the 9 pMMR-CRACs (*P* = 0.002) (Fig. 5A). The proportion of public mutations in the driver genes was also significantly higher in pMMR-JIAC than in pMMR-CRAC (*P* = 0.036) (Fig. 5B). In addition, significant driver gene mutations, such as *ARID2* and *TP53*, arose at early stages in pMMR-JIAC (Fig. 4D). These results suggest that the phylogenetic trees of pMMR-JIAC conform more strongly to a "long trunk-short branches" pattern than those of pMMR-CRAC, likely due to stronger selective pressure.

We inferred the degree of subclonal selection based on the distribution of VAFs and the MATH score [10,34]. The frequencies of clonal and subclonal peaks were similar for pMMR-JIAC and pMMR-CRAC. However, neutral tails were significantly less frequent for pMMR-JIAC (5.0 % vs 56.8 %, *P* < 0.001) (Figs. 5C and D). In addition, the MATH scores were significantly lower for pMMR-JIAC than for pMMR-CRAC (median: 33.538 vs 47.970, *P* < 0.001) (Fig. 5E). These results indicated that pMMR-JIACs have higher VAF peaks than pMMR-CRACs, which leads to lower ITH, possibly due to stronger or more continuous subclonal selection in pMMR-JIACs. Finally, the analysis of the evolutionary histories of two patients with dMMR-JIACs (FJ06 and HM07), as described above, revealed consistent phylogenetic trees with those of the "long trunk-short branches.” The distribution of VAFs and the MATH scores of the dMMR-JIACs indicated higher VAF peaks than those of pMMR-CRACs, which lead to lower ITH (Supplementary Figs. S4A-E). This finding suggests that pMMR-JIACs and dMMR-JIACs are more commonly affected by stronger and more continuous subclonal selection than pMMR-CRACs.

Fig. 5F summarizes the evolutionary histories in this study. According to the recently proposed model [9], most patients with early pMMR-CRAC had findings consistent with the "short trunk-long branches" phylogenetic tree and higher VAF peaks. In contrast, most patients with advanced pMMR-CRAC presented findings consistent with the "long trunk-short branches" phylogenetic tree, a result of Darwinian evolution, and lower VAF peaks, as a result of neutral evolution. In other words, pMMR-CRAC maintains ITH by shifting the evolutionary principle drastically from Darwinian to neutral evolution. Based on our results, however, the advanced pMMR-JIACs findings were more consistent with the “long trunk-short branches” phylogenetic tree due to more involvement of Darwinian evolution and had higher VAF peaks, due to less involvement of neutral evolution. This finding suggests that JIACs in the jejunoileum undergo stronger and more continuous subclonal selection, even at advanced stages, than CRACs in the colorectum, which leads to lower ITH without a shift in the evolutionary principle (i.e., both Darwinian evolution and neutral evolution are present).

## Discussion

Due to the rarity of JIAC, large-scale studies focusing solely on JIAC have not been reported. In this study, we collected 52 JIAC cases, comprising 55 tumors, and successfully identified various critical characteristics of JIAC related to clinicopathological factors, protein expression, prognosis, genetic abnormality, and evolutionary history through comprehensive immunohistochemical and multi-regional WES analyses. Our key findings are as follows: First, the molecular evolutionary process of JIAC differs from that of CRAC. Second, JIAED is a novel histological subtype of JIAC. Third, dMMR-JIAC predominantly exhibits loss of MSH2/MSH6. Fourth, *TP53* and *ARID2* mutations are common driver gene mutations in JIAC. Fifth, high MUC1 expression and low Cyclin D1 expression are independent poor prognostic factors for JIAC. These findings make a very significant contribution to our understanding of JIAC carcinogenesis, which has remained largely unknown.

The most fundamental and significant discovery of our study is that the evolutionary history of JIAC differs from that of CRAC. The evolutionary history of CRAC has been elucidated by Sottoriva *et al.* [8,10] and Saito *et al.* [9]. Early CRAC forms ITH from several subclones, with significant driver mutations and high VAF. However, these mutations are subsequently swept through Darwinian evolution, with the surviving subclones gradually facing various restrictions as the tumor grows. Concurrently, passenger mutations with low VAF that are not affected by these restrictions accumulate neutrally since the early stages (neutral evolution). As a result, advanced CRAC forms ITH from passenger mutations, even in the surviving subclones. These evolutionary dynamics are described by phylogenetic trees and VAF distributions: early CRAC typically has a “short trunk-long branches” phylogenetic tree with high VAF peaks, whereas advanced CRAC has a “long trunk-short branches” phylogenetic tree with low VAF peaks. The findings for JIAC and CRAC in this study (8 patients with 40 pMMR-JIAC tumor samples and 9 patients with 37 pMMR-CRAC tumor samples) indicated that pMMR-JIAC predominantly has a "long trunk-short branches" phylogenetic tree structure, with approximately 90 % of driver mutations located within the trunk. This finding indicates that pMMR-JIAC shares evolutionary tree characteristics with advanced CRAC and reflects the impact of Darwinian evolution. Nevertheless, pMMR-JIAC had high VAF peaks and a low MATH score, leading to lower ITH and suggesting that pMMR-JIAC also shares characteristics with early CRAC, still under Darwinian evolution. Furthermore, our analysis revealed that the phylogenetic trees and VAF distributions of two patients with 11 dMMR-JIAC tumor samples were similar to those of pMMR-JIAC rather than pMMR-CRAC. It is well-established that dMMR tumors are more susceptible to immune cell attack than pMMR tumors because of their high immunogenicity [41]. The similarity of the evolutionary processes of dMMR-JIAC and pMMR-JIAC, but not of dMMR-JIAC and pMMR-CRAC, indicates that JIAC forms a TME in the jejunoileum that differs from that of the colorectum or JIAC has high immunogeneicity.

The present study identified JIAED, a novel histological subtype of JIAC. In gastrointestinal cancers, diagnostic biomarkers such as SALL4 and Glypican 3 are used to identify "gastric adenocarcinoma or CRAC with enteroblastic differentiation (GAED or CRAED)," but JIAED has not been reported previously. Using the same diagnostic biomarkers, we found that 28.8 % of JIAC cases are JIAED. Given that the prevalence of CRAED is 1.5 % in the present study and 4.0 % in other studies [42], it can be concluded that JIAED is more common than CRAED. Furthermore, our investigation revealed that JIAED has the following distinctive characteristics: (a) SALL4-positive JIAED is frequently associated with the expression of neuroendocrine marker Chromogranin A, which may be compatible with the previous case report of CRAED with neuroendocrine expression [35]. (b) Glypican 3 is highly immunogenic and known as a promising target for immunotherapy [43], which may be related to our evolutionary findings in JIAC, and (c) JIAED is more likely to occur in jejunum than in ileum.

Several other findings about JIAC were uncovered as follows. First, driver gene mutations, particularly *TP53* and *ARID2* mutations, were frequently observed in JIAC. In contrast with CRAC, which commonly demonstrates *APC* and *KRAS* mutations, these genetic alterations were less frequent in JIAC, indicating that JIAC is less dependent on the canonical Wnt signaling pathway. Comparison of our pMMR-JIAC data with data from previous SBC studies [3,4] showed consistently frequent *TP53* mutations and rare *APC* mutations, but the frequent *ARID2* mutations represent a novel finding in the present study. They are correlated to enteroblastic differentiation and may affect carcinogenesis and molecular evolutionary history of pMMR-JIAC [11]. Second, MSH2/MSH6 loss was more frequently observed in dMMR-JIAC than in dMMR-CRAC. dMMR-CRAC commonly exhibits MLH1/PMS2 loss due to MLH1 promoter hypermethylation and *BRAF* V600E mutation [37,44], and these were also found in our immunohistochemical results for dMMR-CRAC. In contrast, MSH2/MSH6 loss was the predominant feature in our dMMR-JIAC cases, with no *BRAF* V600E mutations. Furthermore, *MSH2* mutations were detected in all three patients analyzed by WES. This suggests that dMMR-JIAC may have a different carcinogenesis from dMMR-CRAC. Third, high MUC1 and low Cyclin D1 expressions were identified as independent poor prognostic factors for patients with JIAC. These markers have also been reported as poor prognostic factors for CRAC [45,46], suggesting that they may play similar roles in JIAC.

In conclusion, our comprehensive immunohistochemical and multi-regional WES analyses revealed various distinctive characteristics of JIAC, especially from the standpoint of evolutionary process, pathological subclassification, genetic and immunohistochemical features, and prognostic factors. We believe that our findings would lead to better management and treatment strategies for patients with JIAC in the future.

**Data availability**

The data generated in this study are available within the article and its supplementary data files. The raw data for CRACs used for evolutionary analysis are partly available from the SRA database (ID: SRP252694). The software used is available from their respective websites. All other raw data can be obtained upon reasonable request from the corresponding author.

## Funding

This work was supported in part by a Grant-in-Aid from the Japan Society for the Promotion of Science (20K07445 to K.S. and 24K02249 to H.K. and K.S.) and HUSM Grant-in-Aid (to R.I.).

## Ethics approval and consent to participate

The Institutional Review Board of Hamamatsu University School of Medicine (HUSM) approved our study (No. 20-011 and 23-348), which was conducted in accordance with the Declaration of Helsinki.

## CRediT authorship contribution statement

**Rei Ishikawa:** Conceptualization, Data curation, Formal analysis, Investigation, Methodology, Software, Visualization, Writing – original draft, Writing – review & editing. **Hidetaka Yamada:** Supervision. **Hirotomo Saitsu:** Methodology, Resources, Software, Supervision. **Ryosuke Miyazaki:** Resources, Validation. **Juri Takahashi:** Resources, Validation. **Rino Takinami:** Resources, Validation. **Satoshi Baba:** Supervision. **Mitsuko Nakashima:** Supervision. **Moriya Iwaizumi:** Investigation. **Satoshi Osawa:** Investigation. **Hideya Kawasaki:** Investigation. **Yoshifumi Arai:** Resources. **Yoshiro Otsuki:** Resources. **Hiroshi Ogawa:** Resources. **Hiroki Mori:** Resources. **Fumihiko Tanioka:** Conceptualization, Resources. **Shioto Suzuki:** Resources. **Kazuyo Yasuda:** Resources. **Makoto Suzuki:** Resources. **Haruhiko Sugimura:** Conceptualization, Project administration, Resources, Supervision. **Kazuya Shinmura:** Data curation, Funding acquisition, Methodology, Project administration, Resources, Supervision, Writing – review & editing.

## Declaration of competing interest

The authors have no conflict of interest.

## Appendix Supplementary materials

Image, application 1

Image, application 2

Image, application 3

Image, application 4

Image, application 5

Image, application 6
