# Supplementary material for: Immunohistochemical and molecular evolutionary features of jejunoileal adenocarcinoma unveiled through comparative analysis with colorectal adenocarcinoma
Source: Neoplasia. 2025 May 21;66:101180. doi: 10.1016/j.neo.2025.101180 (PMC12148733; doi:10.1016/j.neo.2025.101180)
Supplement: Supplementary file 6 [file mmc6.docx]

**Supplementary Figures**

**Immunohistochemical and Molecular Evolutionary Features of Jejunoileal Adenocarcinoma Unveiled Through Comparative Analysis with Colorectal Adenocarcinoma**

Rei Ishikawa, Hidetaka Yamada, Hirotomo Saitsu, Ryosuke Miyazaki, Juri Takahashi, Rino Takinami, Satoshi Baba, Mitsuko Nakashima, Moriya Iwaizumi, Satoshi Osawa, Hideya Kawasaki, Yoshifumi Arai, Yoshiro Otsuki, Hiroshi Ogawa, Hiroki Mori, Fumihiko Tanioka, Shioto Suzuki, Kazuyo Yasuda, Makoto Suzuki, Haruhiko Sugimura, Kazuya Shinmura


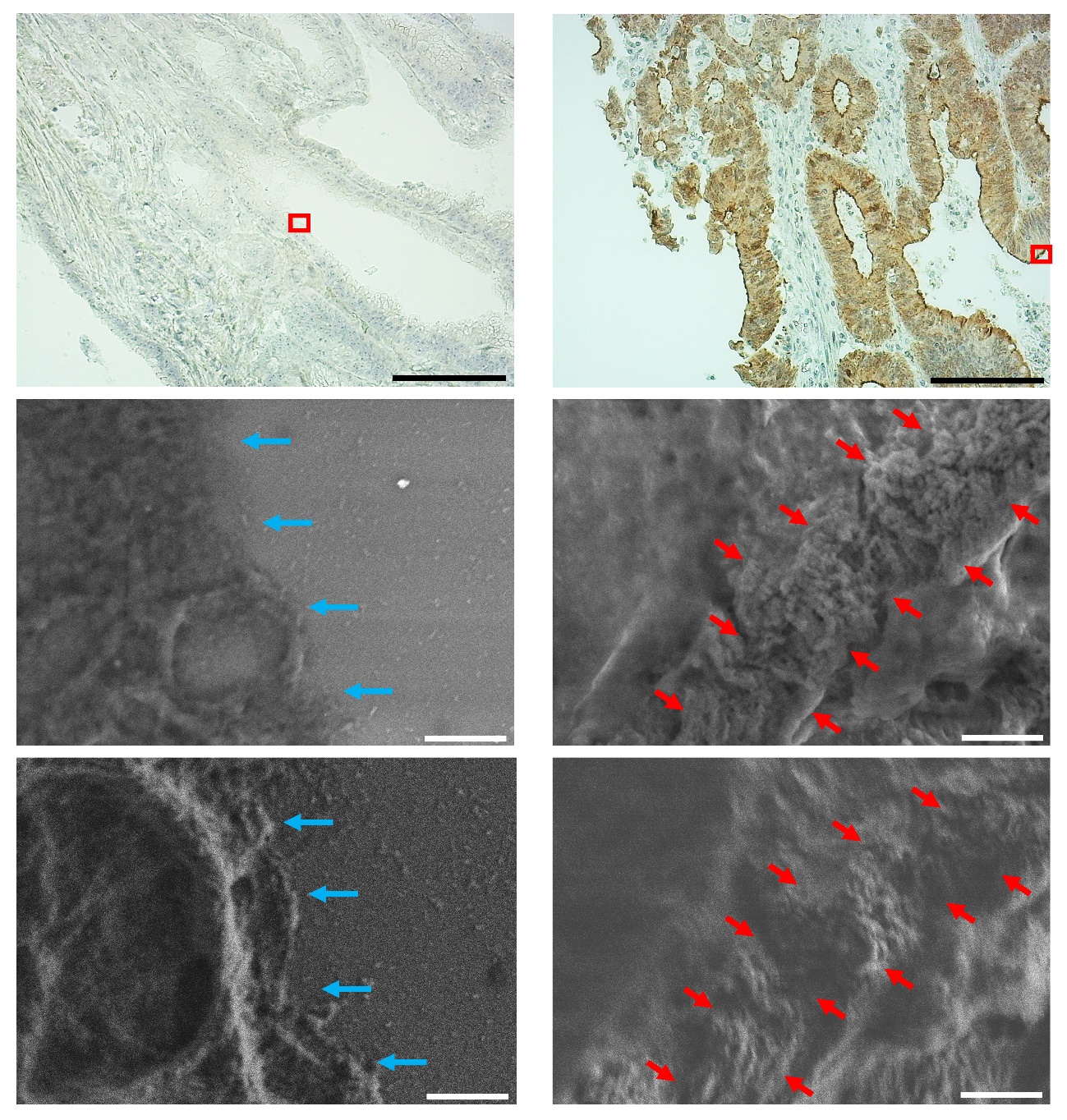


**Supplementary Figure S1. Observation of microvilli of JIAC (left) and CRAC (right) using FE-SEM analysis with the NanoSuit-CLEM method.** The sections used for villin-1 immunohistochemistry were used for the FE-SEM observation. Images were captured using an YAGBSE detector and SE detector. The images of the immunohistochemical slide (top), YAGBSE (middle), and SE (bottom) are shown. The middle and bottom panels correspond to the area of the red square in the top panel. The three-dimensional microvilli are clearly shown in CRAC, but not in JIAC. Red arrows indicate microvilli of CRAC, and blue ones indicate apical surface of JIAC. Scale bar: 100 µm (top); 1 µm (middle and bottom).


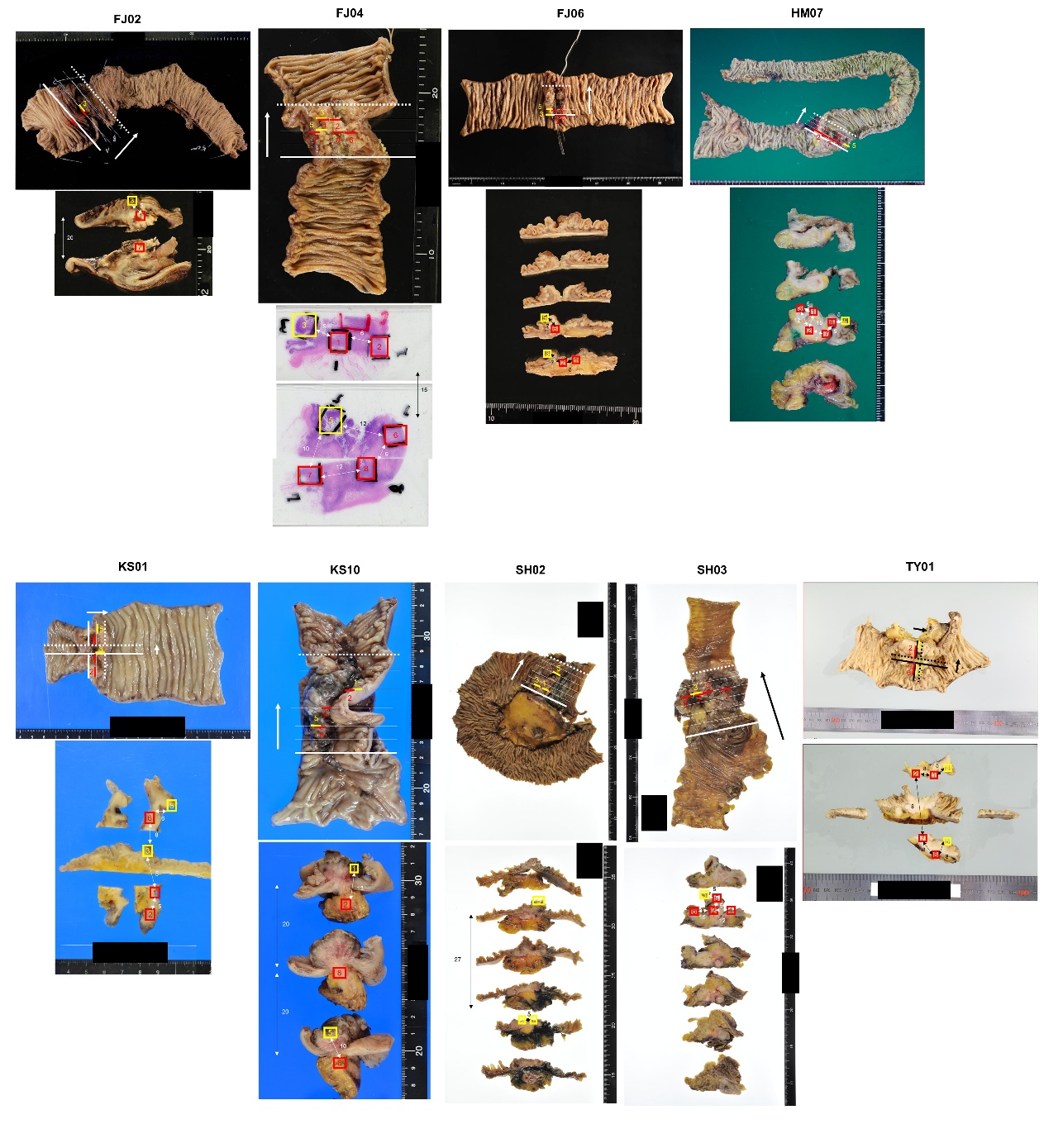


**Supplementary Figure S2. Maps of the sampling locations of JIAC.** Seven patients with pMMR-JIAC (patient ID: FJ02, FJ04, KS01, KS10, SH02, SH03, and TY01) and two patients with dMMR-JIAC (patient ID: FJ06 and HM07) are included. The upper panel depicts the section lines, which are approximately 10 mm apart. Following sectioning, each sample was tilted at right angles in the direction of the arrow. The sampling location is indicated by yellow or red lines, and a serial number is added to each location. Yellow indicates that the sample was taken from the mucosal to the submucosal layer, whereas red indicates that the sample was taken from a layer deeper than the muscularis propria. The lower panel also displays the sampling location on the section surface. As previously noted, yellow and red indicate the same in the upper panel. Two closely spaced sampling locations are connected by a double-headed arrow, and the distance (mm) is shown on the cut surface. For FJ04, the sampling locations are indicated on the hematoxylin-eosin (H-E) slide glasses. The maps were constructed using the same methodology as **Fig. 4A**, which depicts the sampling locations of a pMMR-JIAC (patient ID: FJ01).


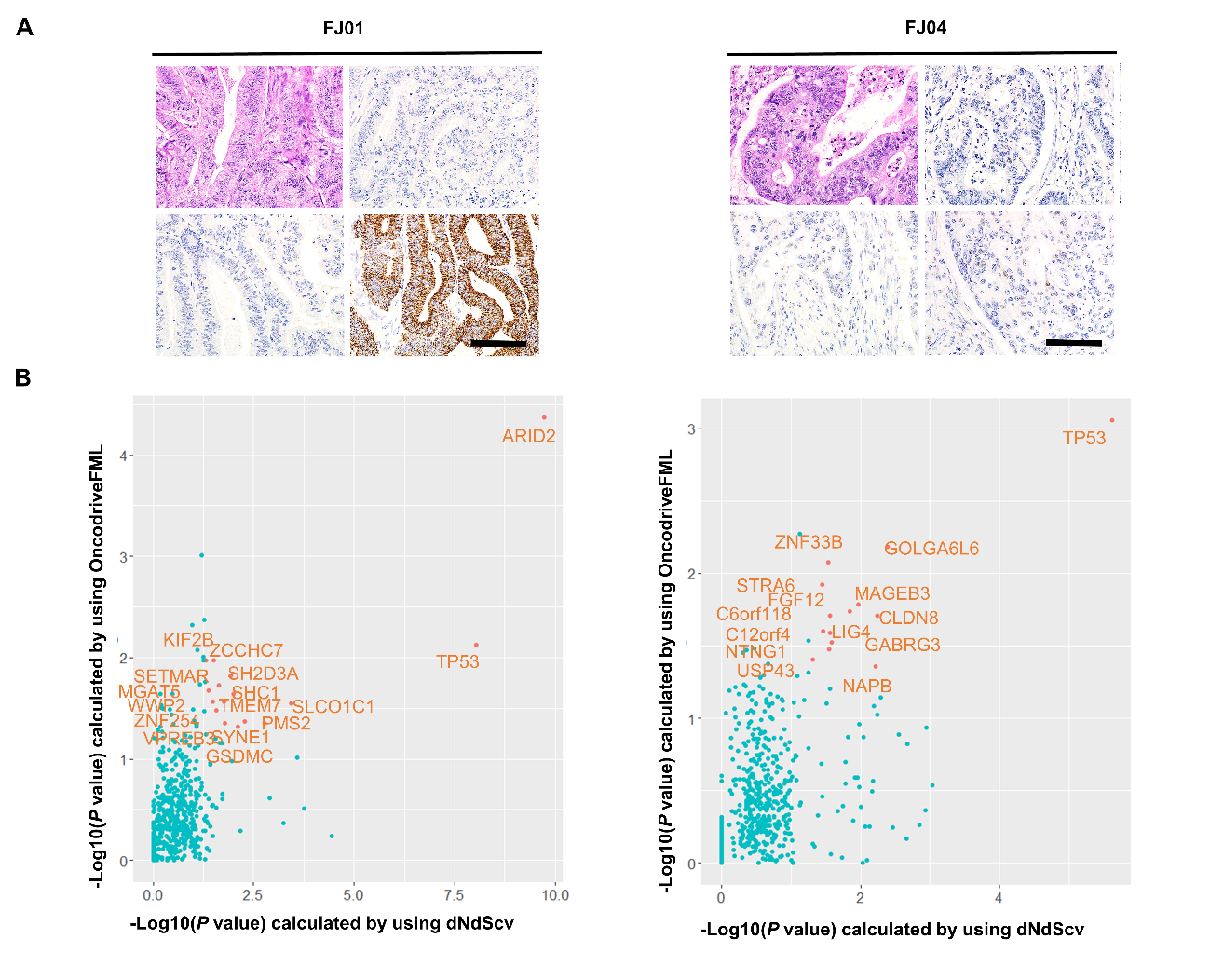


**Supplementary Figure S3. Immunohistochemical and genomic features of JIAED.** Experimental results are compared between four patients with JIAED (left panels) and four patients with JIAC without enteroblastic differentiation (right panels). All 8 JIACs were mismatch repair proficient. **(A)** Representative hematoxylin-eosin (H-E) staining image (top left) and immunohistochemical images of SALL4 (bottom left), AFP (top right), and glypican 3 (bottom right). Note that one patient with JIAED (patient ID: FJ01) showed strong expression of glypican 3, which is one of the enteroblastic differentiation markers, whereas a one patient with JIAC without enteroblastic differentiation (patient ID: FJ04) did not show expression of any of the three enteroblastic differentiation markers. Scale bar, 100 µm. **(B)** Identification of driver genes in JIAED compared with JIAC without enteroblastic differentiation using dNdScv and OncodriveFML. Total mutations of each 20 tumor samples were subjected to dNdScv or OncodriveFML to search for candidate genes with cancer driver mutations. Significance threshold (*P* = 0.05) corresponds to -log_10_(*P-*value) = 1.301. Genes with significant detection in both dNdScv and OncodriveFML were selected as candidate driver genes (orange font). Among them, *ARID2* gene was the most significant driver gene, which was specific to JIAED.


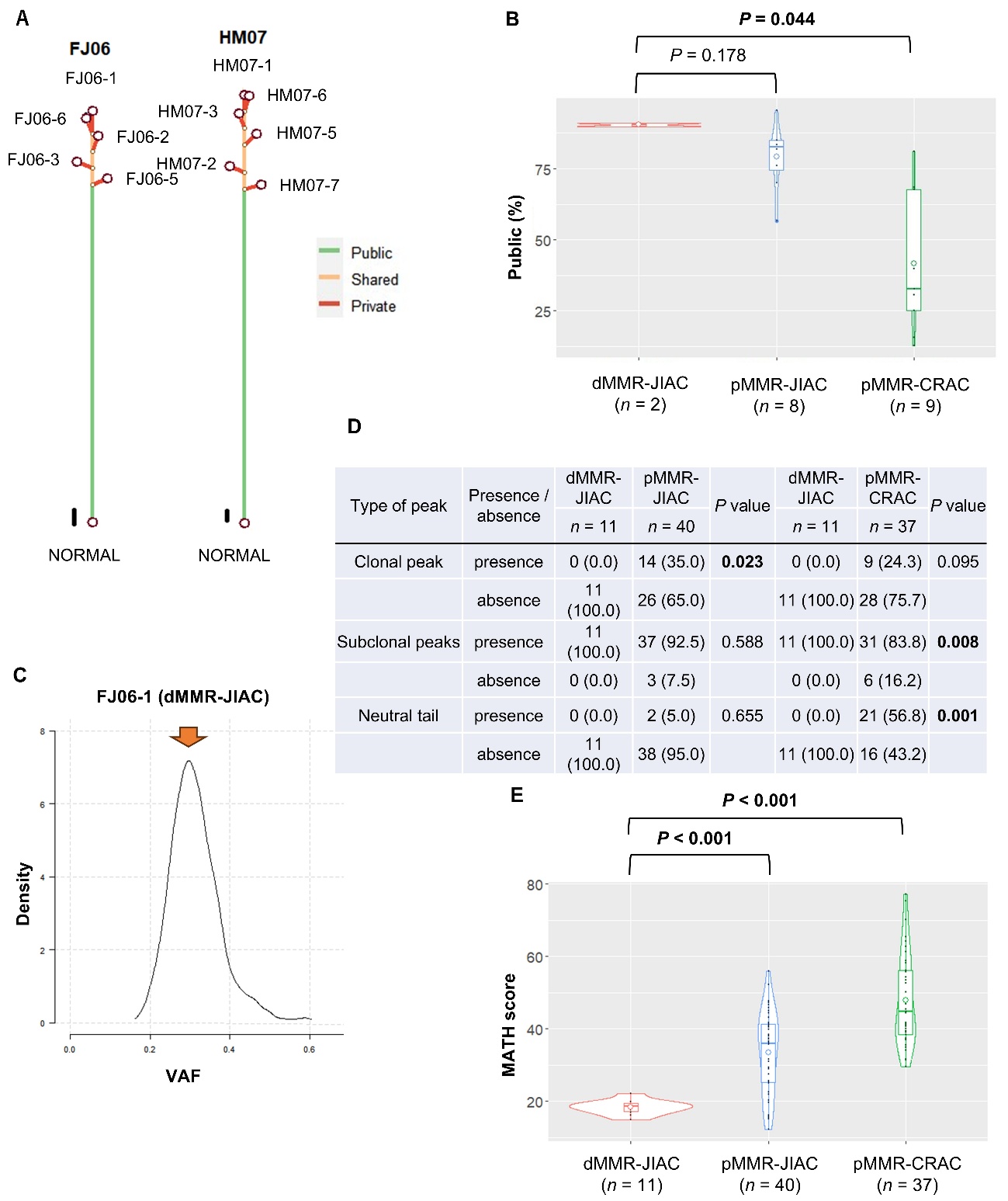


**Supplementary Figure S4. Molecular evolutionary characteristics of dMMR-JIAC.** Two patients with dMMR-JIAC (FJ06 and HM07) underwent multi-regional WES analysis followed by molecular evolutionary analysis. **(A)** Phylogenetic trees of two patients with dMMR-JIAC (FJ06 and HM07). All somatic point mutation data, consisting of base substitutions and small insertions/deletions, were subjected to PyClone-vi, and CCFs per tumor were estimated. Phylogenetic trees were then constructed by neighbor-joining method using the CCFs data and MesKit. Mutations are classified into three groups: public (observed in all multi-regional samples; green), private (observed in only one sample; red), shared (neither public nor private; yellow). The phylogenetic trees shapes of the two dMMR-JIACs are categorized as "long trunk - short branches" (i.e., public mutations predominate). Scale bar, 100 mutations. **(B)** Comparison of the proportion of public mutations among all mutations observed in phylogenetic trees between dMMR-JIAC, pMMR-JIAC, and pMMR-CRAC. The proportions of 2 dMMR-JIACs, 8 pMMR-JIACs, and 9 pMMR-CRACs were analyzed using violin boxplots. The violin plot shows the relative frequency of each value represented by dots, while the boxplot shows the median and interquartile range. The white circle indicates the mean. The proportion of public mutations was significantly different between dMMR-JIAC and pMMR-CRAC (median: 90.4% vs. 41.5%; Mann-Whitney *U*-test; *P* = 0.044). **(C)** Representative data of the VAF distribution of dMMR-JIAC plotted using the Mclust method. The data were obtained from one patient with dMMR-JIAC (sample ID: FJ06-1). The peak (density > 2.0; orange arrow) in the VAF distribution was classified as a subclonal peak (VAF = 0.15-0.40). **(D)** Comparison of the proportion of peaks in the VAF distribution between dMMR-JIAC, pMMR-JIAC, and pMMR-CRAC. Peak data were analyzed by Pearson's chi-square test among 11 tumor samples derived from 2 dMMR-JIACs, 40 tumor samples derived from 8 pMMR-JIACs, and 37 tumor samples derived from 9 pMMR-CRACs. Note that neutral tail were significantly less frequent in dMMR-JIAC than in pMMR-CRAC. Bold font, *P* < 0.05. **(E)** Comparison of the distribution of MATH scores based on VAF data between dMMR-JIAC, pMMR-JIAC, and pMMR-CRAC. The distribution of MATH scores in 11 tumor samples derived from 2 dMMR-JIACs, 40 tumor samples derived from 8 pMMR-JIACs, and 37 tumor samples derived from 9 pMMR-CRACs were analyzed by violin boxplots. The distribution of MATH scores was significantly different between dMMR-JIAC and pMMR-JIAC (median: 18.317 vs 33.538; Mann-Whitney *U*-test; *P* < 0.001) and between dMMR-JIAC and pMMR-CRAC (median: 18.317 vs 47.970; Mann-Whitney *U*-test; *P* < 0.001).
